# Molecular detection of *Rickettsia* species in ectoparasites collected from two southern provinces of Cambodia

**DOI:** 10.1371/journal.pntd.0012544

**Published:** 2024-09-30

**Authors:** Didot Budi Prasetyo, Jodi M. Fiorenzano, Daliya Nop, Nin Noch, Boren Huot, Sokly Mom, Sitha Prum, Visal Chhe, Sokha Dul, Vireak Heang, Satharath Prom, Ju Jiang, Allen L. Richards, Christina M. Farris, Jeffrey C. Hertz

**Affiliations:** 1 Vysnova Partners, LLC, Alexandria, Virginia, United States of America; 2 U.S. Naval Medical Research Unit INDO PACIFIC, Sembawang, Singapore; 3 AC Investment Co., Ltd., Phnom Penh, Cambodia; 4 U.S. Naval Medical Research Unit INDO PACIFIC, Phnom Penh, Cambodia; 5 Department of Health, Ministry of National Defence, Phnom Penh, Cambodia; 6 Naval Medical Research Center, Silver Spring, Maryland, United States of America; THAILAND

## Abstract

Arthropod-borne rickettsioses comprise a wide variety of subtypes that are endemic in Cambodia, but there remains very little data on the geographic distribution of the pathogens or their vectors. Surveys were conducted in Koh Kong and Preah Sihanouk Provinces between September 2017 and June 2018 to collect ectoparasites from peridomestic animals and the environment using dragging and flagging methods. Collected ectoparasites were sorted and identified morphologically, then pooled by species, host, and location for molecular detection using *Rickettsia* genus- and species-specific qPCR and/or multilocus sequence typing (MLST) assays. A total of 14,254 ectoparasites were collected including seven new locality records. *Rickettsia* species were detected in 35.5% (174/505) of the pools screened representing 3,149 randomly selected ectoparasites from the total collected. *Rickettsia asembonensis* was detected in 89.6% (147/164) of *Rickettsia*-positive flea pools and 3.6% (6/164) of the flea pools were positive for both *R*. *asembonensis* and *Rickettsia felis*. *Candidatus* Rickettsia senegalensis from *Ctenocephalides orientis* fleas and *Rickettsia* sp. close to *Rickettsia japonica* and *Rickettsia heilongjiangensis* from *Haemaphysalis* ticks were identified by MLST. This appears to be the first report of these new ectoparasite records and rickettsial species in southern Cambodia, suggesting a potential health risk to military and civilians in this region.

## Author summary

Rickettsiae are Gram-negative bacteria that cause acute undifferentiated febrile illness in humans. These pathogens are associated with arthropods and primarily transmitted by ectoparasites such as ticks, fleas, and mites. In Cambodia, rickettsial diseases have been documented in local patients, travelers and military personnel. However, the lack of entomological studies and awareness of these diseases hindered our comprehensive understanding of these pathogens and their arthropod vectors. Our manuscript provides a description of ectoparasite diversity and sheds light on their potential role in rickettsial disease transmission. Through molecular techniques, we uncover the prevalence and diversity of *Rickettsia* species in ectoparasites collected from various animal hosts in Koh Kong and Preah Sihanouk province. *Rickettsia asembonensis*, *Rickettsia felis*, and *Candidatus* Rickettsia senegalensis were all identified from *Ctenocephalides orientis* fleas, while *Rickettsia* species closely related to *Rickettsia japonica*, *Rickettsia heilongjiangensis*, and *Rickettsia raoultii* were found from *Haemaphysalis* ticks. Additionally, *Rickettsia* were also detected in *Heterodoxus spiniger* lice and Laelapidae mites, although we were unable to characterize the species. Insights into this pathogen-vector-host interface will better inform healthcare professionals, leading to more accurate diagnoses and increased awareness for better management of these diseases.

## Introduction

Rickettsiae are arthropod-borne obligate intracellular Gram-negative bacteria that cause acute undifferentiated febrile illness in humans, with endemic and hyper-endemic focal areas, globally [1]. They are divided into four genetically distinct groups: spotted fever group (SFG), typhus group (TG), transitional group (TSG) and ancestral group (AG) [2]. Members of the scrub typhus group orientiae (STGO) previously belonged to genus *Rickettsia* but were later reclassified into genus *Orientia* due to molecularly and antigenically distinct characteristics [3]. Within the aforementioned rickettsial groups, the severity of the diseases is strongly dependent on the causative species and geographical region [4]. For example, Rocky Mountain spotted fever (RMSF) caused by *Rickettsia rickettsii* in the Americas is considered the most dangerous rickettsial disease and, when left untreated, can lead to organ failure [5].

Ectoparasites such as ticks, lice, fleas, and mites are arthropod vectors responsible for maintaining rickettsiae in natural environments. Human pathogenic rickettsiae can be flea-borne such as *Rickettsia typhi* (TGR) and *Rickettsia felis* (TSGR), tick-borne such as *R*. *rickettsii* and *Rickettsia conorii* (SFGR), mite-borne such as *Orientia tsutsugamushi* (STGO) or louse-borne such as *Rickettsia prowazekii* (TGR). Humans are accidental hosts of ticks, mites, and fleas, which may attach during outdoor activities or close contact with animal hosts. When humans host these ectoparasites, they may acquire infections through bites or exposure to infectious fluids or feces [6,7]. Companion animals, such as domestic cats and dogs, are also susceptible to infection with these pathogens and can serve as potential disease reservoirs, increasing the risk of human disease [8,9].

Rickettsial diseases are the second most common cause of non-malarial febrile illness in Southeast Asia (SE Asia) posing substantial public health risks among rural populations, plantation or forest workers, and refugee populations, as previously noted from cases of individuals living near the Thailand–Myanmar border [10–13]. Throughout Cambodia, rickettsial diseases have been documented in local patients, travelers, and military personnel with undifferentiated febrile illness [14–18]. Additionally, surveillance conducted by the United States Naval Medical Research Unit INDO PACIFIC (NAMRU IP; formerly NAMRU-2) in Cambodia between 2015–2016, revealed that 13.4% of 1,638 human sera samples collected tested positive for antibodies against at least one rickettsial diseases [19]. Of these, 0.98% (16/1,638), 4.33% (71/1,638), and 0.85% (14/1,638) of paired acute and convalescent samples demonstrated seroconversion/4-fold rise in antibody titer against STGO, TGR, and SFGR antigens, respectively, indicating that a substantial proportion of the Cambodian population has been exposed to rickettsial diseases.

Tick and flea-borne rickettsioses comprise a wide variety of endemic subtypes in SE Asia [20], but, due to the lack of robust entomological surveillance and limited infrastructure for accurate diagnosis these subtypes are not well understood in Cambodia. As such, the true burden of rickettsial diseases in Cambodia remains unknown. To further understand the potential risk of rickettsial diseases in Cambodia, NAMRU IP, in collaboration with the Cambodian Department of Health (DoH) within Ministry of National Defence, conducted an ectoparasite surveillance study in two southern provinces, Koh Kong and Preah Sihanouk. Key aims of the study were to characterize the vector diversity and prevalence of rickettsiae among vector populations in this region.

## Materials and methods

### Ethical statement

The study protocol was reviewed and approved by the Naval Medical Research Center (NMRC) Institutional Animal Care and Use Committee (IACUC) in compliance with all applicable federal regulations governing the protection of animals and research, IACUC Protocol Number EXM-17-15-NAMRU-2.

### Study sites

**Fig 1:**
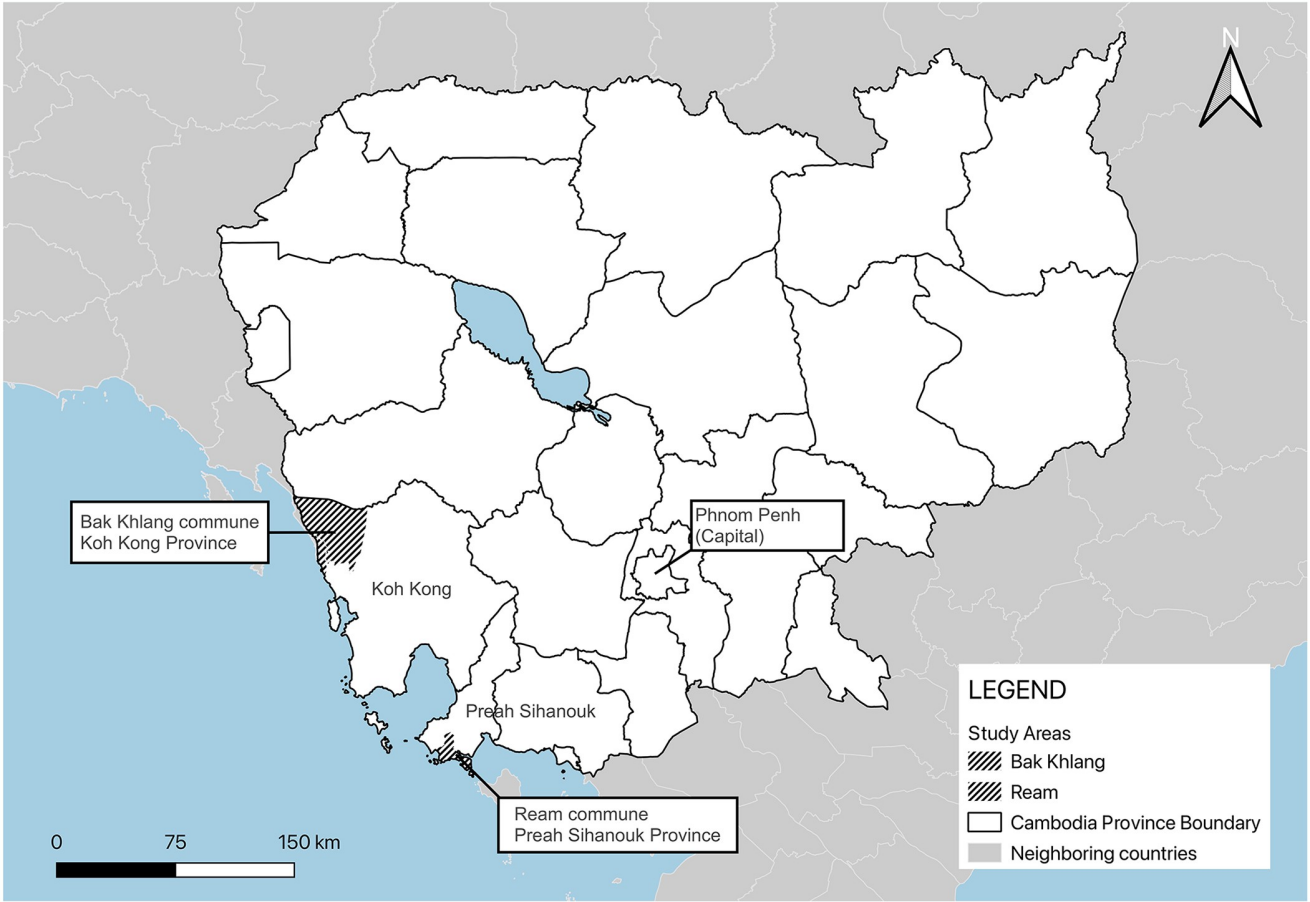
Collection sites in Koh Kong and Preah Sihanouk provinces. Maps were generated using the free, open-source QGIS software. Subnational administrative boundary shapefiles for Cambodia and neighboring countries are available for download from The Humanitarian Data Exchange (https://data.humdata.org/dataset/).

The study was conducted in two southern provinces of Cambodia: Preah Sihanouk and Koh Kong (Fig 1), where institutional collaborations were well-established and there was significant interest in assessing the risk of rickettsial diseases as part of force health protection for the Royal Cambodian Armed Forces and public health of residents in these regions. In Preah Sihanouk, ectoparasites were collected at Ream commune, Prey Nob district. In Koh Kong province, ectoparasites were collected at Pak Khlang commune, Mondul Seima district. The study areas included households of military personnel, and GPS coordinates of the ectoparasite collection points were not recorded upon request. Both collection sites were classified as typical rural Cambodian environments, however, their surroundings differed substantially. Collection sites in Koh Kong province were surrounded by or near forested areas, while sites in Preah Sihanouk were more developed (urban-like).

### Specimen collection

Ectoparasites were collected either directly from hosts or from environment using drags/flags between September 2017 to June 2018. Households were selected in coordination with local authorities based on the possession of domestic animals and willingness to participate in the study. In each household visited, cats, dogs, chickens and cattle were inspected with the support and assistance of the animal owner. A thorough visual inspection was conducted to check for the presence of ectoparasites on all body parts for a duration of up to 30 minutes per animal. The total number of animals inspected per household was limited to the number of animals that could be examined within the one-hour visit. Inspections were performed carefully to prevent discomfort. Feral and domesticated stray animals were not examined. In rare instances, inspections were also performed opportunistically on deceased game animals, if present during the survey. Synanthropic rats and mice were captured using a combination of collapsible Sherman (7.62×8.89×22.86 cm) and Tomahawk live traps (15.2x15.2x48.26 cm). A total of five Sherman and five Tomahawk traps (spaced 10 meters apart) were set up per day around the visited houses starting at 6 pm, using dried fish as baits. The traps were checked the following morning, and any captured rats and mice were euthanized prior to ectoparasite collection.

Fleas, mites and lice were removed from animals using a combination of brushing and combing craniocaudally. A white plastic tray was positioned underneath to collect ectoparasites from the comb and brush. Ticks were removed from animals using fine tip forceps by carefully grasping the tick’s capitulum and gently pulling straight up until the tick released itself from the host’s skin. Collected ectoparasite specimens were preserved in tubes containing 70% ethanol and transferred to the NAMRU IP laboratory in Phnom Penh, the capital of Cambodia.

The collection of free-living ticks was conducted in tandem with house-to-house animal inspections. A set of transects (up to 1 km each) were created at the surrounding vegetations. The collection was performed by walking the transects while dragging a 1 m x 1 m white fabric over the vegetation between 9 to 11 am. The collector stopped every 10 m to remove collected ticks from the fabric using a lint roller (or manually). Collected ticks were transferred to zip lock bags and labelled, then placed inside a cooler box until transported to the NAMRU IP laboratory where they were removed from the lint roller, counted, and preserved in 70% ethanol.

### Morphological identification

In the laboratory, ectoparasites were identified morphologically to the finest taxa possible using appropriate identification keys for each group of ectoparasite [21–40]. Ectoparasite specimens were then pooled by species, life stage, sex, host, and location. Due to the large number of ectoparasites collected, a sub-sample from each host was used for *Rickettsia* screening without distinguishing by life stage or sex. A total of 505 pools (280 from Koh Kong and 225 from Preah Sihanouk) were randomly selected with 1 to 10 individual per pool for ticks and 1 to 20 individual per pool for smaller-bodied ectoparasites such as fleas, lice, and mites.

### DNA extraction and molecular analysis

With the exception of mites, due to their size, ectoparasite specimens were cut in half prior to DNA extraction. Half of the specimens were used for molecular analysis, while the remaining half were archived in the NAMRU IP freezers for future studies. DNA was extracted using Qiagen DNA mini kit (Qiagen, Germany) following manufacturer’s instructions with some modification to the sample lysis step. Ectoparasites were ground using a disposable pestle in 180 μL of ATL buffer and 20 μL Proteinase K. The mixtures were then incubated for 2 hours at 56° C. Hard and larger-bodied ectoparasites such as ticks and cattle lice were incubated overnight to ensure proper digestion of the tissues. Following incubation, 200 μl AL buffer was added to the mixture and further incubated at 70° C for 10 min. 100 μL absolute ethanol was added to the sample, and the mixture was transferred to a QIAamp DNA spin column. The sample was then centrifuged at 8,000 rpm for 1 min. The supernatant was discarded, and the column was washed with 500 μl each of buffer AW1 and AW2. The DNA was eluted with 50 μl of AE elution buffer and kept at −20°C prior to molecular screening.

**Fig 2:**
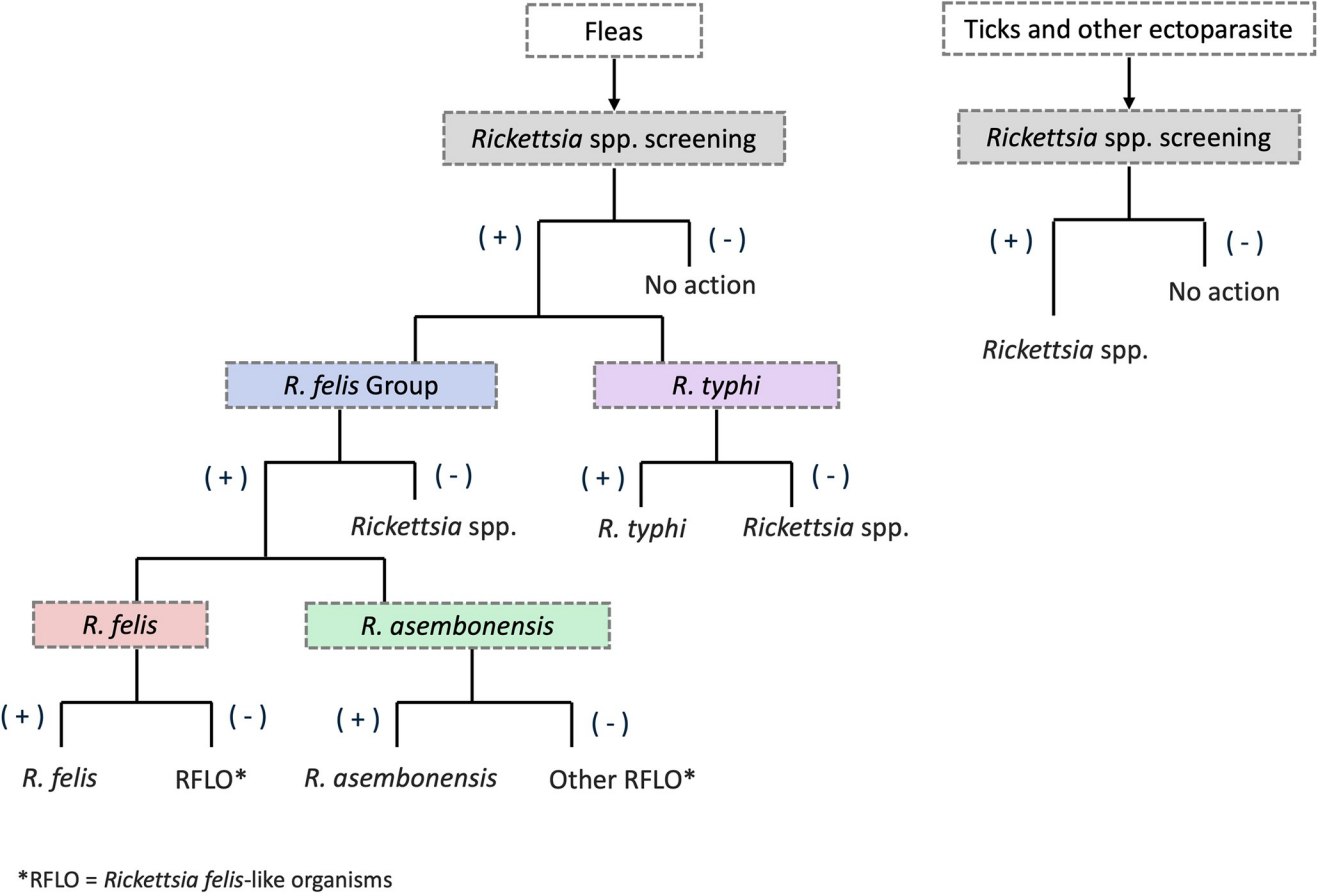
Algorithm for detection and identification of ectoparasite-borne rickettsioses by qPCR assays.

Ectoparasites were initially screened with *Rickettsia* genus-specific qPCR assay (Rick17B) targeting the 17 kDa gene region [41]. *Rickettsia*-positive flea samples were subjected to further qPCR assays specifically designed to detect flea-borne rickettsiosis [42–44]. All primers and probes used in these assays are listed in Table 1 and the PCR algorithm is shown in Fig 2. qPCR was performed using GoTaq qPCR Master Mix (Promega, United States) and run on an Applied Biosystems 7500 Real-Time PCR platform. The annealing temperature and the concentrations of the primers, the probe, MgCl_2_, and CXR Reference Dye were optimized. Each 25 μL qPCR reaction contained 0.4 μL CXR Reference Dye and different concentrations of primers and probes: For *R*. *typhi*, 0.2 μM of primer, 0.3 μM probe and 5mM of MgCl_2_; For *R*. *felis* Group, 0.5 μM of primer, 0.4 μM of probe and 5mM of MgCl_2_; For *R*. *felis*, 0.5 μM of primer, 0.4 μM of probe, and 7 mM of MgCl_2_; For *R*. *asembonensis*, 0.3 μM of Primer, 0.7 μM of probe, and 7 mM of MgCl_2_. A total of 2 μL of template DNA was used in each reaction. The cycler parameters included incubation for 2 min at 50°C, initial denaturation for 10 min at 95°C and 45 cycles of denaturation for 15 s at 95°C, and annealing/elongation for 30 s at 60°C.

**Table 1:** *Rickettsia* qPCR primer and probe sets.

qPCR Assay	Primer/Probe Name	Sequence (5’-3’)
*Rickettsia* spp.	R17K128F2	GGGCGGTATGAAYAAACAAG
	R17K238R	CCTACACCTACTCCVACAAG
	R17K202TaqP	FAM-CCGAATTGAGAACCAAGTAATGC-TAMRA
*R*. *typhi*	RT557F	TGGTATTACTGCTCAACAAGCT
	RT678R	CAGTAAAGTCTATTGATCCTACACC
	RT640Bprobe	TET-CGCGATCGTTAATAGCAGCACCAGCATTATCGCG-DAB
*R*. *felis* Group	RF1396F	ACCCAGAACTCGAACTTTGGTG
	RF1524R	CACACCCGCAGTATTACCGTT
	RF1448BP-FAM	FAM-CGCGACTTACAGTTCCTGATACTAAGGTTCTTACAGGTCGCG-BHQ-1
*R*. *felis*	Rfel_phosp_MBF	GCAACCATCGGTGAAATTGA
	Rfel_phosp_MBR	GCCACTGTGCTTCACAAACA
	Rfel_phosp_MBP	FAM-CCGCTTCGTTATCCGTGGGACC-TAMRA
*R*. *asembonensis*	Rasem2828F	CACACTTAGCGGCGGTATTC
	Rasem2939R	AAGTTGTTATAGTCTGTAGTAAACG
	Rasem2893BFAM	FAM-CCGCAGCTCCAATACCTTCGCCTAAGCCATATGCGG-BHQ-1

*Rickettsia*-positive samples not identified by species-specific qPCR were subjected to PCR amplification of the 17 kDa, *ompA*, *ompB*, *rrs*, *sca4*, and *gltA* genes to identify the *Rickettsia* species using multilocus sequencing typing (MLST) [43,45–47]. The primer sequences for the genes amplification are listed in S1 Table. Amplified PCR products were purified using the QIAquick PCR purification kit and sequenced. The sequences obtained were analyzed and compared to those available in GenBank using Basic Local Alignment Search Tool (BLAST). The *Rickettsia* species were characterized based on highest percent identity to the reference sequences.

### Data analysis

The prevalence of ectoparasites on hosts was calculated by dividing the number of infested hosts by the number of total hosts inspected. Mean intensity (MI) was expressed as total number of ectoparasites per number of infested hosts. Prevalence and MI were calculated using Qpweb software Version 1.0.15 [48]. The density of free-living ticks was expressed as the number of ticks per 100 m^2^. Infection rates for pooled ectoparasites were calculated using PooledInfRate (version 4.0) Microsoft Excel Add-in. Since the pool size varied, the bias-corrected Maximum Likelihood Estimation (MLE) method with 95% confidence intervals (CI) was used and expressed as the MLE of infected ectoparasite per 100 ectoparasites.

## Results

A total of 570 domestic animals were inspected: dogs (N = 369, 64.7%), cattle (N = 66, 11.6%), murid rodents (N = 122, 21.4%), poultry (N = 9, 1.6%), cat (N = 1, 0.2%), tortoise (N = 1, 0.2%), and monitor lizards (N = 2, 0.4%). Domestic dogs were the most frequently encountered host during the collection. A total of 8,745 ectoparasites were collected in Koh Kong province, comprising ticks (N = 6,765, 77.4%), fleas (N = 1,259, 14.4%), lice (N = 389, 4.4%), and mites (N = 332, 3.8%). In Preah Sihanouk, a total of 5,509 ectoparasites were collected, comprising ticks (N = 3,951, 71.7%), fleas (N = 809, 14.7%), lice (N = 674, 12.2%), and mites (N = 75, 1.4%).

Ectoparasite species identified in the study and their respective hosts are presented in Table 2. The prevalence of ectoparasite infestations were varied among the hosts. The highest numbers of ectoparasite infestations recorded included: *Rhipicephalus microplus* sensu lato (s.l.) collected from cattle in Koh Kong (42/47, Prevalence = 0.894) and *Rhipicephalus linnaei* (tropical lineage of *Rh*. *sanguineus* s.l.) collected from dogs in Preah Sihanouk (103/170, Prevalence = 0.606). Mean Intensity (MI) was found highest in mites *Laelaps echidninus* collected from murid rodents in Koh Kong (234/7, MI = 33.4), followed by *Rh*. *microplus* s.l. tick collected from cattle in Preah Sihanouk (251/8, MI = 31.4).

**Table 2:** Ectoparasite species collected from animals in Koh Kong and Preah Sihanouk.

Location	Host species (n inspected)	Ectoparasite species	n host infested	Total ectoparasite	Prevalence	MI (95% CI)
Koh Kong	*Canis familiaris* (199)	*Ctenocephalides orientis*	94	1,177	0.472	12.5 (10.6–15.1)
		*Ctenocephalides felis*	1	2	0.005	2
		*Echidnophaga gallinacea*	3	47	0.015	15.7 (1–29.7)
		*Dermacentor* spp. nymph	17	54	0.085	3.18 (1.99–5.06)
		*Haemaphysalis asiatica* ^*+*^	1	1	0.005	1
		*Haemaphysalis hystricis* ^*+*^	3	9	0.015	3 (1–4)
		*Haemaphysalis papuana*	7	11	0.035	1.57 (1–2.14)
		*Haemaphysalis* spp.	6	10	0.03	1.5 (1–1.67)
		*Rhipicephalus linnaei*	27	199	0.136	7.37 (4.35–13)
		*Rhipicephalus microplus* s.l.	6	29	0.03	4.83 (2.17–8.5)
		*Heterodoxus spiniger* ^*+*^	45	389	0.226	8.64 (6.42–12.5)
	*Felis catus* (1)	*Dermacentor* spp. nymph	1	2	1	2
	*Bos indicus* (47)	*Dermacentor* spp. nymph	2	2	0.043	1
		*Rhipicephalus microplus* s.l.	42	425	0.894	10.1 (7.71–14.6)
		*Haemaphysalis* sp.	1	1	0.021	1
	*Gallus gallus domesticus* (3)	*Echidnophaga gallinacea*	2	25	0.667	12.5 (2–12.5)
	*Varanus salvator* (2)	*Amblyomma varanense*	2	6	1	3 (1–3)
	Geoemydidae Tortoise (1)	*Amblyomma geoemydae*	1	1	1	1
	Muridae Rodents (52)	*Haemaphysalis bandicota* ^*+*^	2	16	0.038	8 (2–14)
		*Dermacentor* spp. nymph	5	15	0.096	3 (1.2–4.8)
		*Xenopsylla cheopis*	3	8	0.058	2.67 (1–4)
		*Laelaps nutalli* ^*+*^	3	98	0.058	32.7 (14–43)
		*Laelaps echidninus* ^*+*^	7	234	0.135	33.4 (19.3–52.3)
		*Total*		2,761		
Preah Sihanouk	*Canis familiaris* (170)	*Ctenocephalides orientis*	39	801	0.229	20.5 (12.6–38.2)
		*Rhipicephalus linnaei*	103	3,099	0.606	30.1 (24.2–45.3)
		*Heterodoxus spiniger* ^*+*^	37	659	0.218	17.8 (11.6–27.7)
	*Bos indicus* (19)	*Haematopinus quadropertusus*	1	1	0.053	1
		*Rhipicephalus microplus* s.l.	8	251	0.421	31.4 (17.1–50.6)
	Muridae Rodents (70)	*Xenopsylla cheopis*	5	8	0.071	1.6 (1–2.2)
		*Haemaphysalis* spp.	1	3	0.014	3
		*Laelaps echidninus* ^*+*^	11	71	0.157	6.45 (2.24–15.4)
		Unidentified Gamasid *mites*	1	4	0.014	4
		*Hoplopleura oenomydis* ^*+*^	1	1	0.014	1
	*Meleagris* sp. (2)	*Lipeurus* spp.	2	13	1	6.5 (2–6.5)
		*Total*		4,911		

Note: ^+^New record in Cambodia

Prevalence = the proportion of infested hosts among all examined hosts

MI (Mean Intensity) = mean number of ectoparasites found on infested hosts

A total of 5,984 free-living ticks were collected during 9 km of cloth dragging in Koh Kong and 598 ticks were collected during 5 km of cloth dragging in Preah Sihanouk. This comprised 68.4% and 10.8% of the total ectoparasite collection in Koh Kong and Preah Sihanouk, respectively. The tick collected in both sites consisted almost exclusively of *Rhipicephalus (Boophilus)* spp. larvae, with addition of *Haemaphysalis* and *Dermacentor* larvae found only in Koh Kong (Table 3). The density of questing *Rhipicephalus (Boophilus)* spp. larvae was higher in Koh Kong sites (66 larvae/100m^2^) than in Preah Sihanouk sites (7 larvae/100 m^2^).

**Table 3:** Number of ticks collected by dragging method in Koh Kong and Preah Sihanouk.

Location	Distance dragged (m)	Species (Life stage)	Total No.	Density
Koh Kong	9,040	*Dermacentor* spp. (Larvae)	3	0.03/100 m^2^
		*Haemaphysalis* spp. (Larvae)	20	0.2/100 m^2^
		*Rhipicephalus* spp. (Larvae)	5,961	66/100 m^2^
Preah Sihanouk	5,450	*Rhipicephalus* spp. (Larvae)	598	7/100 m^2^

As shown in Table 4, *Rickettsia* spp. were detected in 124 pools of fleas (*Ctenocephalides orientis*), five pools of ticks (two *Haemaphysalis hystricis*, one *Haemaphysalis papuana*, one *Haemaphysalis bandicota*, one *Rh*. *linnaei*), and two pools of lice (*Heterodoxus spiniger*) in Koh Kong. In Preah Sihanouk, *Rickettsia* spp. were detected in 37 pools of fleas (*C*. *orientis*), two pools of ticks (*Rh*. *linnaei*), three pools of lice (*H*. *spiniger*), and one pool of mites (*L*. *echidninus*). In both study sites, none of the ticks collected by dragging tested positive for *Rickettsia* spp.

**Table 4:** Presence of *Rickettsia* in ectoparasites collected from Koh Kong and Preah Sihanouk.

Location	Host species / Method	Ectoparasite species	n pool tested	n positive pool			
				*Rickettsia* spp.	RFLO^+^	*R*. *asembonensis*	*R*. *felis and* *R*. *asembonensis*
Koh Kong	*Canis familiaris*	*Ctenocephalides orientis*	126	124	7	115	2
		*Echidnophaga gallinacea*	4	-	-	-	-
		*Dermacentor* spp. nymph	19	-	-	-	-
		*Haemaphysalis hystricis*	3	2	-	-	-
		*Haemaphysalis papuana*	9	1	-	-	-
		*Haemaphysalis* spp.	3	-	-	-	-
		*Rhipicephalus linnaei*	11	1	-	-	-
		*Rhipicephalus microplus* s.l.	8	-	-	-	-
		*Heterodoxus spiniger*	29	2	-	-	-
	*Felis catus*	*Dermacentor* spp. nymph	1	-	-	-	-
	*Bos indicus*	*Dermacentor* spp. nymph	1	-	-	-	-
		*Rhipicephalus microplus* s.l.	39	-	-	-	-
		*Haemaphysalis* spp.	1	-	-	-	-
	*Gallus gallus domesticus*	*Echidnophaga gallinacea*	3	-	-	-	-
	*Varanus salvator*	*Amblyomma varanense*	2	-	-	-	-
	Geoemydidae Tortoise	*Amblyomma geoemydae*	1	-	-	-	-
	Muridae Rodents	*Haemaphysalis bandicota*	2	1	-	-	-
		*Dermacentor spp*. nymph	3	-	-	-	-
		*Xenopsylla cheopis*	3	-	-	-	-
		*Laelaps nutalli*	2	-	-	-	-
		*Laelaps echidninus*	4	-	-	-	-
	Dragging method	*Dermacentor* spp. larva	2	-	-	-	-
		*Haemaphysalis* spp. larva	3	-	-	-	-
		*Rhipicephalus* spp. larva	1	-	-	-	-
		Total	280	131	7	115	2
Preah Sihanouk	*Canis familiaris*	*Ctenocephalides orientis*	38	37	1	32	4
		*Rhipicephalus linnaei*	121	2	-	-	-
		*Heterodoxus spiniger*	34	3	-	-	-
	*Bos indicus*	*Haematopinus quadropertusus*	1	-	-	-	-
		*Rhipicephalus microplus* s.l.	14	-	-	-	-
	Muridae Rodents	*Xenopsylla cheopis*	4	-	-	-	-
		*Laelaps echidninus*	8	1	-	-	-
		*Hoplopleura oenomydis*	1	-	-	-	-
	Dragging method	*Rhipicephalus* spp. larva	4	-	-	-	-
		Total	225	43	1	32	4

^+^RFLO = *Rickettsia felis*-like organism, excluding *R*. *asembonensis*

At least five species of *Rickettsia* were identified from Koh Kong sites. *Rickettsia asembonensis* and *R*. *felis* were identified through species specific PCR assays, while *Candidatus* Rickettsia senegalensis, one *Rickettsia* spp. genetically close to *Rickettsia japonica* and *Rickettsia heilongjiangensis*, and one *Rickettsia* spp. close to *Rickettsia raoultii* were all characterized through the multiple sequence analysis in the BLASTn database (Table 5). Phylogenetic analyses of these MLST gene sequences are shown in S1–S5 Figs, and the methodology for constructing the maximum likelihood phylogenetic tree is detailed in S1 Methodology.

**Table 5:** Molecular detection of rickettsiae and BLAST analysis of the gene sequences from ticks and fleas.

Location	Ectoparasite Host	Target genes	Query cover (%)	Percent Identity (%)	Molecular identification by BLAST
Koh Kong	*C*. *orientis*	17kDa	91	98.74	*R*. *asembonensis* (OP974448.1)
		gltA	98	100	*Ca*. R. senegalensis (MZ851165.1)
		16S	100	99.7	*Ca*. R. senegalensis (OM311169.1)
		sca4	100	100	*Ca*. R. senegalensis (KT304220.1)
		ompB	100	100	*Ca*. R. senegalensis (KT304219.1)
		ompA	-	-	-
	*C*. *orientis*	17kDa	93	100	*Ca*. R. senegalensis (KU167052.1)
		gltA	94	99.72	*Ca*. R. senegalensis (MZ851165.1)
		16S	100	99.75	*Ca*. R. senegalensis (OM311169.1)
		sca4	100	100	*Ca*. R. senegalensis (KT304220.1)
		ompB	100	100	*Ca*. R. senegalensis (KT304219.1)
		ompA	-	-	-
	*Ha*. *papuana*	17kDa	100	99.07	*R*. *raoultii* (MH932034.1)
		gltA	100	99.19	*R*. *heilongjiangensis* (PP116514.1)
		16S	100	99.47	*R*. *japonica* (CP047359.1)
		sca4	100	99.72	*R*. *heilongjiangensis* (MG906668.1)
		ompB	100	100	*R*. *hulinensis* (AY260452.1)
		ompA	100	99.02	*R*. *heilongjiangensis* (AP019863.1)
	*Ha*. *hystricis*	17kDa	100	98.61	*R*. *raoultii* (MH932034.1)
		gltA	100	99.18	*R*. *raoultii* (MF511253.1)
		16S	-	-	-
		sca4	100	100	Uncultured *Rickettsia* sp. (PP548180.1)
		ompB	-	-	-
		ompA	-	-	-
Preah Sihanouk	*C*. *orientis*	17kDa	100	100	*R*. *asembonensis* (MK923744.1)
		gltA	100	100	*R*. *asembonensis* (MN186290.1)
		16S	100	99.75	*R*. *asembonensis* (JN315967.1)
		sca4	100	100	*R*. *asembonensis* (MK923740.1)
		ompB	100	99.69	*R*. *asembonensis* (MK862574.1)
		ompA	-	-	-

Note: "-" indicates failed amplification of the respective gene.

*Rickettsia* species detected from *C*. *orientis* fleas were *R*. *asembonensis* (115/126; 91.2%), both *R*. *asembonensis* and *R*. *felis* (2/126; 1.5%), *Ca*. R. senegalensis (2/126; 1.5%), and another *Rickettsia* spp. for which the genes could not be amplified for MLST (5/126; 3.9%). *Rickettsia* species detected from ticks included a *Rickettsia* spp. genetically close to *R*. *japonica* and *R*. *heilongjiangensis* from *Ha*. *papuana* ticks (1/9; 11.1%) and another *Rickettsia* spp. genetically close to *R*. *raoultii* from *Ha*. *hystricis* ticks (1/3; 33%). *Rickettsia* MLST identifications could not be performed on *rickettsia*-positive *Rh*. *linnaei* ticks (1/11; 9%), *Ha*. *bandicota* ticks (1/2; 50%), and *H*. *spiniger* lice (2/29; 6.8%) due to amplification failures.

In Preah Sihanouk, only two species of *Rickettsia* were identified: *R*. *asembonensis* and *R*. *felis*. *Rickettsia asembonensis* (32/38, 84.2%) and both *R*. *asembonensis* and *R*. *felis* co-occurence (4/38, 10.5%) were detected in *C*. *orientis* fleas. The amplification of genes for MLST was unsuccessful for other *Rickettsia*-positive samples of *Rh*. *linnaei* (2/121, 1.6%), *H*. *spiniger* (3/34, 8.8%), and *L*. *echidninus* (1/8, 12.5%). Consequently, further analysis could not be performed on these samples.

When examined using the *Rickettsia* genus-specific qPCR assay, the infection rate was highest in *C*. *orientis* fleas collected from dogs in Koh Kong (MIR = 81.8) compared to the same species in Preah Sihanouk (MIR = 64). *Rickettsia asembonensis* had the highest infection rate in *C*. *orientis* fleas collected from dogs in Koh Kong (MIR = 41.6) compared to Preah Sihanouk (23.6). The infection rate for *R*. *felis* and *R*. *asembonensis* co-occurrence was highest in *C*. *orientis* fleas collected from dogs in Preah Sihanouk (MIR = 1.4) compared to Koh Kong (MIR = 0.3).

## Discussion

In Cambodia, rickettsial diseases have been documented in travelers and patients with undifferentiated febrile illness, and one of the agents, *Rickettsia felis* has been detected in dog populations [14,15,17,18,49]. Yet, due to limited resources and infrastructure for accurate diagnosis and limited resources for vector studies, the true burden of rickettsial disease in Cambodia remains unknown. Therefore, it is imperative that foundational knowledge characterizing the ectoparasites diversity, ecology, and associated hosts and pathogens in Cambodia is established to properly determine the public health threats from rickettsial diseases.

The results of this research expand the known distribution of ectoparasite species in Cambodia. Two lice species (*H*. *oenomydis* and *H*. *spiniger*), two mite species (*L*. *echidninus* and *L*. *nutalli*), and three tick species (*Ha*. *asiatica*, *Ha*. *bandicota*, and *Ha*. *hystricis*) are new records for the country, despite each of them being well documented in neighboring countries in the region. Indeed, these new local records are indicative of the scant vector data available in Cambodia particularly when considering that all new records were collected from domestic dogs and rodents; two of the most ubiquitous hosts to survey. Moreover, *L*. *echidninus* and *H*. *spiniger* were collected from each survey location likely indicating a wide distribution in the country. The remaining ectoparasite species collected in this survey have been previously documented in Cambodia [49–53].

Like many other countries in SE Asia, the dog ownership in Cambodia is estimated to be high, with dog-to-human ratios 1:3.8 in Kandal and 1:3.3 in Battambang province [54]. Domestic dogs were the most frequently encountered ectoparasite hosts in this survey and were associated with the highest diversity of ectoparasites. The most common ectoparasites collected from dogs were *C*. *orientis* fleas, followed by chewing lice *H*. *spiniger*, and *Rh*. *linnaei* ticks. Unlike dogs, domestic cattle in both study areas were primarily infested with *Rh*. *microplus* s.l. This species complex is a common parasite of cattle and can cause a significant problem in livestock production [55]. Various rickettsial pathogens have been detected from *C*. *orientis*, *Rh*. *linnaei*, *Rh*. *microplus* s.l. in neighboring countries, emphasizing their medically important status in the region [56–65].

More rickettsia-positive ectoparasites in this survey were collected from dogs as compared to other hosts. Of these, 93% of the rickettsia positive samples were pools of *C*. *orientis* fleas. *Ctenocephalides orientis* are the most common flea species collected from companion dogs in Asian countries and are known to bite humans [66–68]. A high prevalence of *R*. *asembonensis* in *C*. *orientis* fleas collected from dogs in both locations of this study was observed. The association of *R*. *asembonensis*, previously referred to as a *R*. *felis*-like organism (RFLO), with *C*. *orientis* fleas have been reported in Malaysia [69], Thailand [70,71], Laos [72], and Vietnam [73].

*Rickettsia asembonensis* has also been detected in other vertebrate hosts including monkeys, wild felidae, and small ruminants [74–76] highlighting the zoonotic risk of spillover. Although this rickettsial species has been detected in human blood samples [77–79], its pathogenicity remains to be determined. Six pools of *C*. *orientis* fleas collected from dogs (two from Koh Kong and four from Preah Sihanouk) with *R*. *asembonensis* were also positive for *R*. *felis*. *Rickettsia felis* is a known human pathogen and domestic cats and dogs are a natural reservoir hosts [9]. Inpankaew *et al*. [49] found that nearly 11% of 101 free-roaming dogs sampled in Preah Vihear province were infected with *R*. *felis*. *Candidatus* R. senegalensis, another RFLO, was detected in *C*. *orientis* fleas from Koh Kong province. The high infection rate of *Rickettsia* spp. found from this flea further highlights the medical importance of this species. No rickettsiae were detected in other flea species from this study.

Although the prevalence was lower compared to fleas, *Rickettsia* species found in ticks were of important significance. *Rickettsia* species closely related to *R*. *japonica*, *R*. *heilongjiangensis*, and *R*. *raoultii* were all detected from *Haemaphysalis* ticks and only from Koh Kong. Each of these species of spotted fever *Rickettsia* are known to be infective to humans and have been found from tick specimens from other SE Asian countries [60,80–85]. Attempts to identify the species of *Rickettsia* from other positive samples, including from the *Rh*. *linnaei* and *Ha*. *bandicota* ticks, the *H*. *spiniger* lice, and the *L*. *echidninus* mites were not successful as the gene sequences required for MLST analysis could not be amplified. This suggest that the DNA concentration might be too low for successful amplification, which hampered the ability to ascertain the identity of rickettsial agents in those ectoparasites.

Importantly, the DNA detection from the whole body of an ectoparasite limits the assessment of infective status or transmission capability due to the uncertainty of whether the detected rickettsial agents are capable to proliferate inside the ectoparasites or are present in the remaining blood meals taken from the host. Another limitation is that the reliance on morphological methods to identify ectoparasite species, which can be difficult for immature stages as shown in this survey. Only 53% of specimens collected here were able to be identified to species. The same issue arises for vertebrate host identification, especially with rodents. In future studies, the utilization of molecular techniques for species identification will greatly enhance and complement traditional morphological identification methods.

Findings from this study expands the foundational knowledge on the vector-pathogen interface between ectoparasites, rickettsial pathogens, and their hosts in Cambodia. The presence of rickettsiae circulating in ectoparasite populations from domestic animals, especially domestic dogs, underline a potential public health risk in this region. Future studies should involve sampling a greater diversity of hosts across a much larger geographical area to provide robust insights into the correlation between ectoparasites, rickettsial agents, and their animal hosts, which will better inform healthcare professionals and lead to more accurate diagnoses.

## Supporting information

S1 Table PCR and Nested PCR Primers for MLST. (XLSX)

S1 Methodology Sequence analysis method. (DOCX)

S1 Fig Maximum Likelihood tree for the partial *gltA* gene of *Rickettsia* species. Sequences from this study are indicated with blue color. The scale-bar represents the number of substitutions per site. Substitution model: HKY+F+G4.
(TIF)

S2 Fig Maximum Likelihood tree for the partial 17 kDa gene of *Rickettsia* species. Sequences from this study are indicated with blue color. The scale-bar represents the number of substitutions per site. Substitution model: TPM2+G4.
(TIF)

S3 Fig Maximum Likelihood tree for the partial *sca4* gene of *Rickettsia* species. Sequences from this study are indicated with blue color. The scale-bar represents the number of substitutions per site. Substitution model: K3Pu+F+G4.
(TIF)

S4 Fig Maximum Likelihood tree for the partial 16S gene of *Rickettsia* species. Sequences from this study are indicated with blue color. The scale-bar represents the number of substitutions per site. Substitution model: K2+G.
(TIF)

S5 Fig Maximum Likelihood tree for the partial *ompB* gene of *Rickettsia* species. Sequences from this study are indicated with blue color. The scale-bar represents the number of substitutions per site. Substitution model: T92+G.
(TIF)

S1 Dataset Ectoparasite collection in Koh Kong and Preah Sihanouk province. (XLSX)

S2 Dataset *Rickettsia* detection on ectoparasites collected from Koh Kong and Preah Sihanouk province. (XLSX)
