# Supplementary material for: Molecular detection of Rickettsia species in ectoparasites collected from two southern provinces of Cambodia
Source: PLoS Negl Trop Dis. 2024 Sep 30;18(9):e0012544. doi: 10.1371/journal.pntd.0012544 (PMC11476676; doi:10.1371/journal.pntd.0012544)
Supplement: S1 Methodology — (DOCX) [file pntd.0012544.s002.docx]

**Supplementary material and method**

**Sequence Analysis**

The sequence reads generated on the ABI 3500 Genetic Analyzer were processed using Sequencher version 5.1. The consensus sequences were then aligned with reference *Rickettsia* sequences retrieved from the GenBank database using ClustalW for multiple alignment. A maximum likelihood phylogenetic tree was constructed using the best-fit nucleotide substitution model with 1000 bootstrap replicates in IQ-TREE version 2.3.5. The phylogenetic trees for *gltA*, 17 kDa, *sca4*, 16S (*rrs*), and *ompB* gene sequences were drawn, modified, and visualized using FigTree version 1.4.4. The phylogenetic tree for the *ompA* gene was not constructed due to lower amplification success.
