# Supplementary figures and images for: Molecular detection of Rickettsia species in ectoparasites collected from two southern provinces of Cambodia
Source: PLoS Negl Trop Dis. 2024 Sep 30;18(9):e0012544. doi: 10.1371/journal.pntd.0012544 (PMC11476676; doi:10.1371/journal.pntd.0012544)

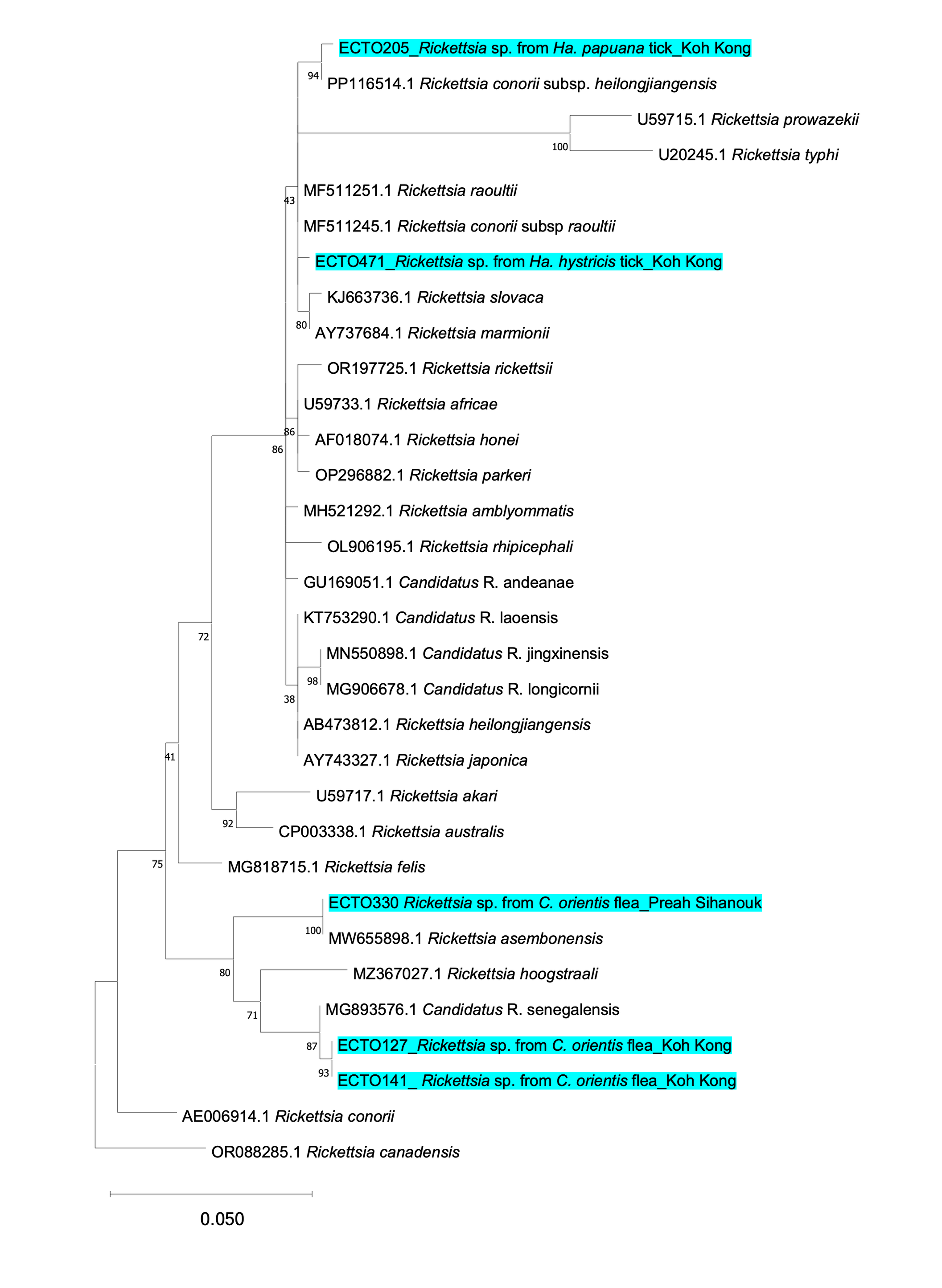

Supplement: S1 Fig — Sequences from this study are indicated with blue color. The scale-bar represents the number of substitutions per site. Substitution model: HKY+F+G4. (TIF) [file pntd.0012544.s003.tif]

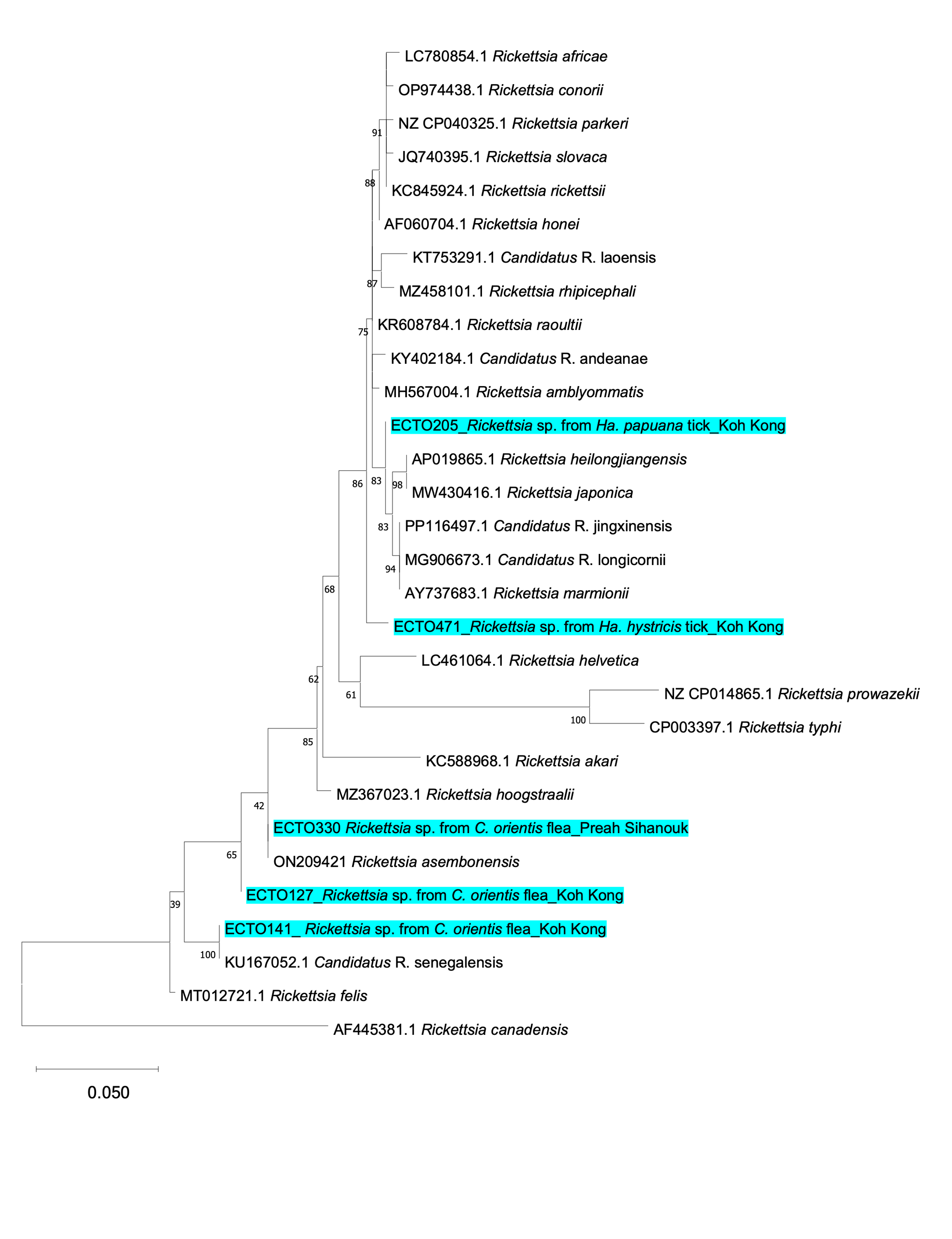

Supplement: S2 Fig — Sequences from this study are indicated with blue color. The scale-bar represents the number of substitutions per site. Substitution model: TPM2+G4. (TIF) [file pntd.0012544.s004.tif]

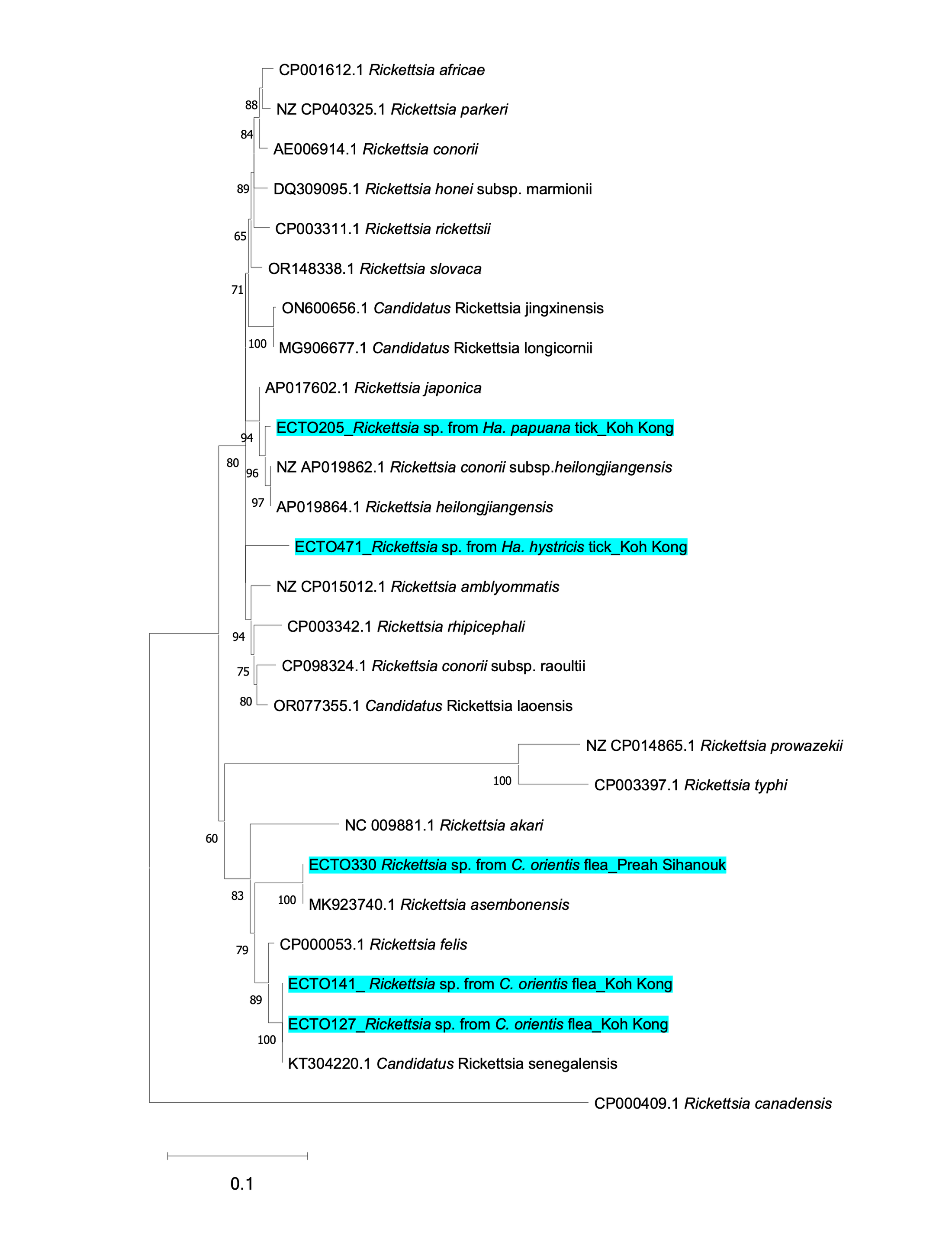

Supplement: S3 Fig — Sequences from this study are indicated with blue color. The scale-bar represents the number of substitutions per site. Substitution model: K3Pu+F+G4. (TIF) [file pntd.0012544.s005.tif]

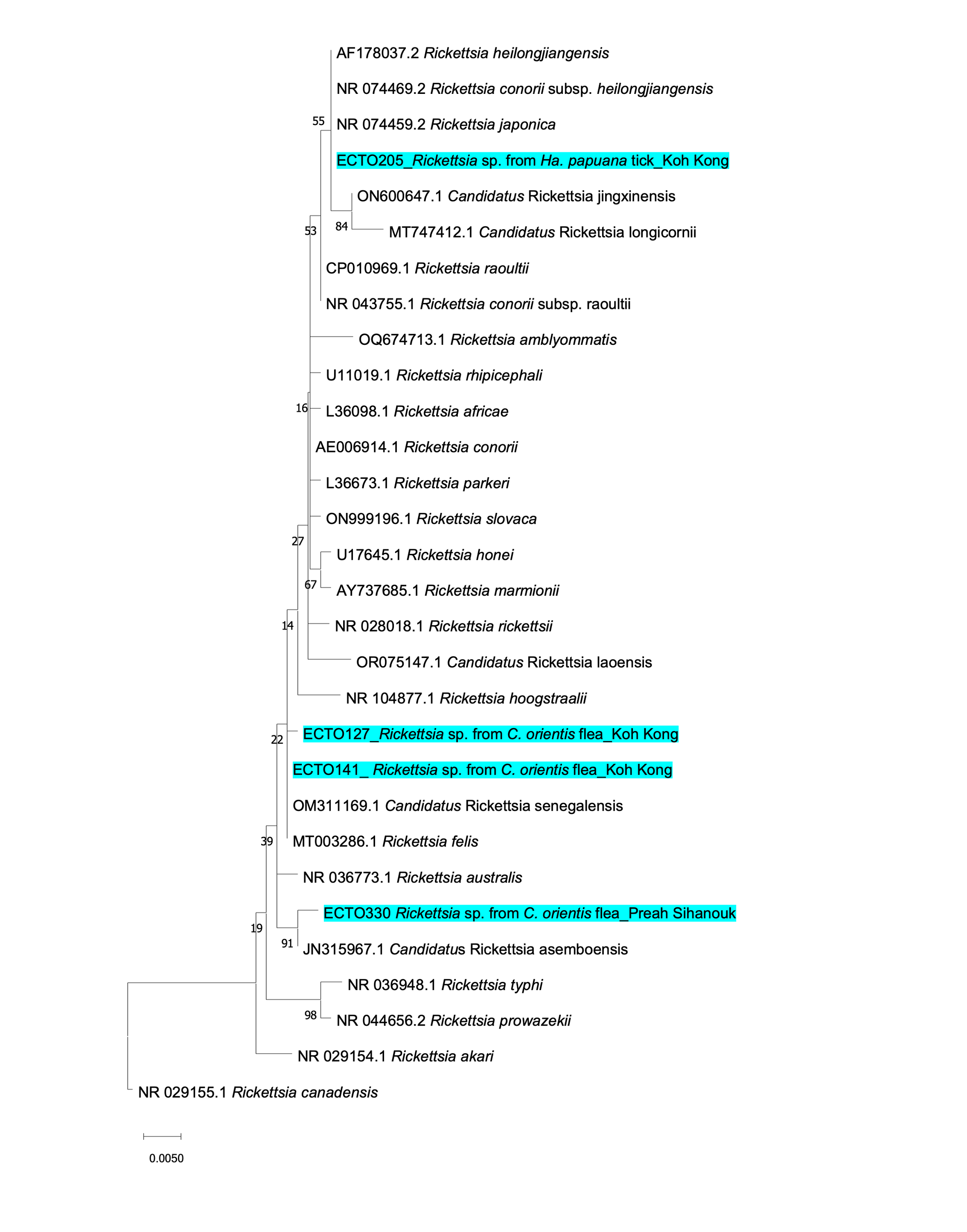

Supplement: S4 Fig — Sequences from this study are indicated with blue color. The scale-bar represents the number of substitutions per site. Substitution model: K2+G. (TIF) [file pntd.0012544.s006.tif]

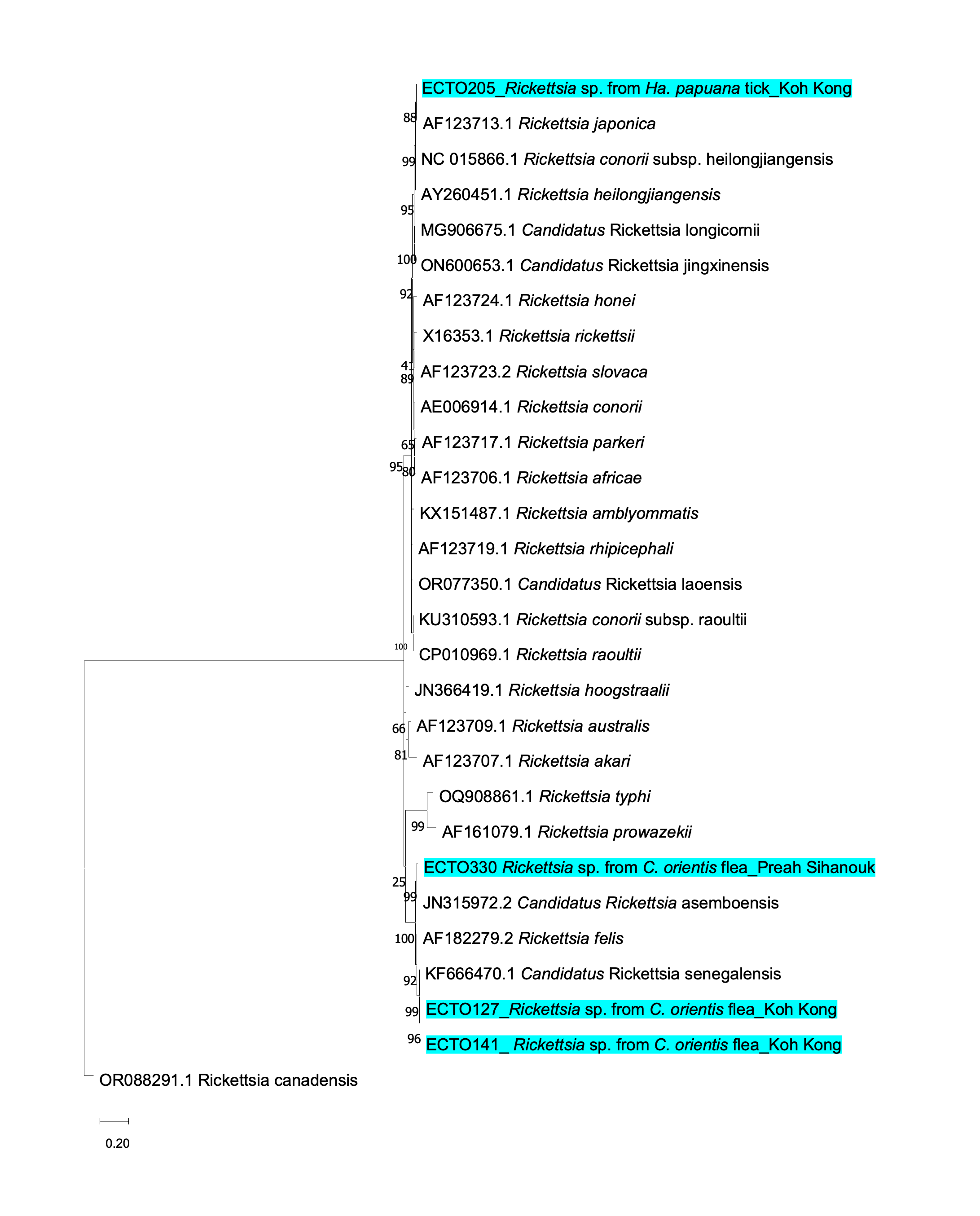

Supplement: S5 Fig — Sequences from this study are indicated with blue color. The scale-bar represents the number of substitutions per site. Substitution model: T92+G. (TIF) [file pntd.0012544.s007.tif]
